# A correlation study of serum tumor markers with systemic lupus erythematosus-associated interstitial lung disease

**DOI:** 10.3389/fmed.2025.1558702

**Published:** 2025-03-18

**Authors:** Xing He, Jiaqi Ji, Ting Zhou, Zeli Luo, Siyu Fang, Xixi Chen, Chi Liu, Haiying Yan, Lu Guo

**Affiliations:** ^1^Department of Pulmonary and Critical Care Medicine, West China Hospital, Sichuan University, Chengdu, China; ^2^State Key Laboratory of Respiratory Health and Multimorbidity, West China Hospital, Sichuan University, Chengdu, China; ^3^Department of Pulmonary and Critical Care Medicine, Sichuan Provincial People’s Hospital, School of Medicine, University of Electronic Science and Technology of China, Chengdu, Sichuan, China; ^4^Department of Critical Care Medicine, Wenjiang District People’s Hospital of Chengdu, Chengdu, Sichuan, China; ^5^Department of Rheumatology and Immunology, Sichuan Provincial People’s Hospital, University of Electronic Science and Technology of China, Chengdu, Sichuan, China; ^6^Department of Nephrology, Sichuan Academy of Medical Science and Sichuan Provincial People’s Hospital, Sichuan Renal Disease Clinical Research Center, University of Electronic Science and Technology of China, Chengdu, Sichuan, China

**Keywords:** systemic lupus erythematosus, interstitial lung disease, tumor markers, biomarkers, organ involvement

## Abstract

**Background:**

Systemic lupus erythematosus-associated interstitial lung disease (SLE-ILD) is a pulmonary manifestation of SLE. Currently, serum biomarkers for early identification of SLE-ILD are lacking. Our study aimed to investigate the correlation and clinical significance of serum tumor markers (TMs) in patients with SLE-ILD.

**Methods:**

We conducted a retrospective analysis of medical records from SLE patients between January 2017 and November 2023. We compared the differences in serum levels of TMs including carcinoembryonic antigen (CEA), carbohydrate antigens (CA125, CA15-3, and CA19-9), squamous cell carcinoma antigen (SCC), cytokeratin-19-fragment (CYFRA21-1), neuron-specific enolase (NSE) and ferritin (FER), between SLE-ILD and SLE patients.

**Results:**

A total of 386 SLE patients were enrolled in this study, comprising 64 individuals with SLE-ILD. Compared with SLE group, SLE-ILD group exhibited higher serum levels of CEA, CA125, CA15-3, CA19-9, SCC, CYFRA21-1, and FER. Multivariate regression indicated that age (OR = 1.038, 95% CI = [1.004, 1.074]), CA15-3 (OR = 1.099, 95% CI = [1.039, 1.162]), and CA19-9 (OR = 1.032, 95% CI = [1.005, 1.059]) were associated factors for SLE-ILD (*p* < 0.05). Serum levels of CA15-3 demonstrated good diagnostic value with an area under the receiver operating characteristic curve (AUC) = 0.72; furthermore, combining age with serum levels of CA19-9 and CA15-3 presented enhanced diagnostic performance as reflected by an AUC = 0.80 (95% CI = [0.73, 0.86]). Serum levels of SCC and CYFRA21-1 moderately positively correlated with serum creatinine levels (*r* = 0.562 and 0.713, respectively).

**Conclusion:**

Serum levels of CA125, CA15-3, and CA19-9 could act as associated markers for SLE-ILD. Serum SCC, CYFRA21-1 and FER levels may also be linked to kidney involvement in SLE-ILD.

## Introduction

Systemic lupus erythematosus (SLE) is a connective tissue disease (CTD) characterized by multisystem damage, which mainly occurs in young women. It often involves the kidneys, hematological systems, skin, and lungs. SLE-associated interstitial lung disease (SLE-ILD) represents lung involvement in SLE and is an important risk factor for death in patients with SLE (1). The impact of SLE on the lungs can manifest in various forms, including pleurisy, pleural effusion, pulmonary embolism, and pulmonary hypertension. ILD is recognized as a pulmonary complication in patients with SLE, and its estimated prevalence ranges from approximately 3–9% (2). The 5-year and 10-year cumulative probabilities of pulmonary damage in SLE patients are 7.6 and 11.6%, respectively (3). In recent years, SLE-ILD has been characterized by progressive fibrosis (4). Some common types of SLE-ILD can be identified using chest high-resolution computed tomography (HRCT), including non-specific interstitial pneumonia (NSIP), usual interstitial pneumonia (UIP), and organizing pneumonia (OP) (5). However, due to the absence of respiratory symptoms and young age at onset, these conditions may be overlooked— resulting in decreased lung function and impaired quality of life. In addition to SLE-ILD, most patients with rheumatoid arthritis-associated interstitial lung disease (RA-ILD) lack clinical symptoms, and there are no optimal tools for early screening and long-term follow-up (6). Therefore, early detection of SLE-ILD is of great significance.

Serum tumor markers (TMs) have long been considered metabolites of tumor tissue, including carcinoembryonic antigen (CEA), carbohydrate antigens (CA125, CA15-3, and CA19-9), cytokeratin-19-fragment (CYFRA21-1) and neuron-specific enolase (NSE). They are widely utilized to assist in tumor diagnosis and evaluate therapeutic effects. Recent studies have demonstrated that some serum TMs may be involved in the occurrence and progression of connective tissue disease-associated interstitial lung disease (CTD-ILD); furthermore, some TMs may correlate with organ involvement and disease activity in patients with SLE (7–9). As a non-invasive screening strategy, the potential value of TMs in the evaluation of SLE-ILD remains unclear. In this study, we analyzed the differences in serum TMs levels between patients with SLE-ILD and those with SLE alone, thereby exploring the correlation between serum TMs and SLE-ILD.

## Materials and methods

### Study methods and participants

In this retrospective study, we included patients who were first diagnosed with SLE between January 2017 and November 2023 at the Department of Rheumatology, Sichuan Provincial People’s Hospital & University of Electronic Science and Technology of China. The study was conducted in accordance with the principles of the Declaration of Helsinki, and was approved by Institutional Review Board of Sichuan Provincial People’s Hospital, University of Electronic Science and Technology of China (Process No. 2016–111). The Ethics Committee of Sichuan Provincial People’s Hospital, University of Electronic Science and Technology of China agreed to exempt patients from written informed consent.

### Eligibility criteria

All included patients were diagnosed in accordance with the 2019 EULAR/ACR diagnostic criteria for SLE (10). The diagnosis of SLE-ILD was based on ILD manifestations by HRCT examinations, such as subpleural reticular opacities, honeycombing, traction bronchiectasis, ground-glass opacities and consolidation. Other secondary causes (lung infectious diseases, increased cardiac load and pulmonary edema, for instance) were systematically excluded. Ultimately, the diagnosis of SLE-ILD was finally confirmed by a multidisciplinary team.

Exclusion criteria were as follows: (1) complications with other CTD; (2) systemic infectious diseases; (3) malignant cancers; (4) pregnancy; (5) immunodeficiency; (6) complications with pulmonary embolism, pleural effusion, and atelectasis; (7) digestive tract diseases; and (8) missing data.

It was taken into consideration that early malignancy was undetectable, which would influence serum TMs levels; therefore, patients diagnosed with malignancies within 1 year of the first diagnosis of SLE-ILD were also excluded.

### Data collection and standards

Data including age, gender, body mass index (BMI), disease course, peripheral white blood cell count (WBC), hemoglobin (HGB), platelets (PLT), Urea, creatinine (Cr), 24 h urinary protein quantity (24hUpr), erythrocyte sedimentation rate (ESR), serum lactate dehydrogenase (LDH), serum albumin, serum creatine kinase (CK), serum immunoglobulins (G, A, M), serum complement (C3, C4), and other laboratory results were obtained from medical records of patients with SLE during their initial hospitalization. The disease course was defined as the interval from the onset of the first clinical symptom associated with SLE.

Serum TMs included CEA, CA125, CA15-3, CA19-9, squamous cell carcinoma antigen (SCC), CYFRA21-1, NSE, and ferritin (FER), all of which were detected by electro-chemiluminescence assays. The reference ranges for each serum tumor marker level are presented as follows: CEA < 3.4 ng/mL, CA125 < 35 U/mL, CA15-3 < 25 U/L, CA19-9 < 27 U/L, SCC < 1.5 ng/mL, CYFRA21-1 < 2.08 ng/mL, NSE <16.3 ng/, FER: male <400 ng/mL, female <150 ng/mL.

All laboratory results were obtained at the first test after admission.

### Sample size calculation

To ensure the reliability of the multivariate analysis, 10 candidate factors were incorporated into the multivariate regression analysis. According to the sample size estimation method, the sample size for each group was 5 × n (n = number of differential factors) (11), resulting in a minimum sample size of 50 cases per group, which was considered statistically significant.

### Statistical analysis

All data were analyzed for normality test by Shapiro–Wilk test. Non-normally distributed data were expressed as median (interquartile range), and the Mann–Whitney *U* test was employed for intergroup differences. Categorical variables were calculated by chi-square test. Univariate logistic regression was performed to identify potential factors associated with SLE-ILD. If the results were statistically significant, multivariate logistic regression was used to identify independent markers of SLE-ILD. A receiver operating characteristic (ROC) curve was used to evaluate the diagnostic value of serum TMs for SLE-ILD, and the area under the ROC curve (AUC) and cut-off values were calculated for each variable. Pearson correlation analysis was executed to explore the correlation between serum TMs levels and other organ functions. SPSS 26.0 software was used and *p* < 0.05 was considered statistically significant.

## Results

### General characteristics of the patients

**Figure 1:**
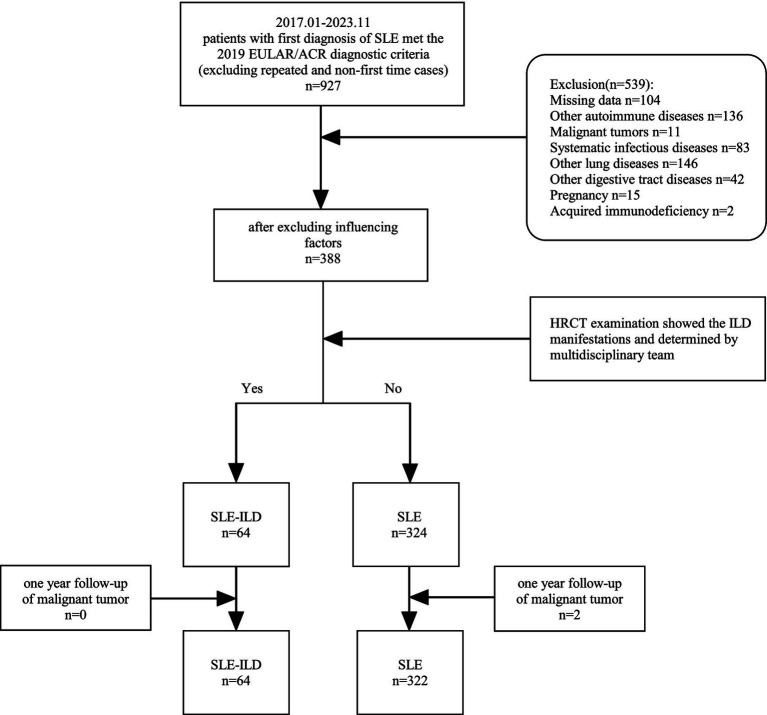
Flowchart of patient enrollment.

We included 386 patients with SLE, among whom 64 were diagnosed with SLE-ILD (Figure 1). None of the enrolled patients were receiving any glucocorticoids, immunosuppressants, or antifibrotic drugs at the time of first admission. In the SLE-ILD group, most patients were female (4 males out of 60 participants), with a median age of 49 years and a median disease course of 60 months (SLE-related antibodies and HRCT imaging findings were shown in Supplementary Table 1). Patients in the SLE-ILD group were older than those in the SLE group (*p* < 0.05); no statistical significances were observed between the two groups regarding the disease course, gender, or BMI. The levels of ESR, LDH, CK and 24hUpr were higher in the SLE-ILD group than in the SLE group (*p* < 0.05). Conversely, the HGB level was lower in the SLE-ILD group than in the SLE group, and there were no statistical differences between the two groups concerning serum levels of WBC, PLT, Cr, Urea, IgG, IgA, IgM, C3, C4 and IgE (Table 1).

**Table 1:** Baseline characteristics of included patients.

	SLE (*n* = 322)	SLE-ILD (*n* = 64)	*Z*/*χ*^2^	*P*
Age (year)	38[29,48]	49[40,60]	−4.444	<0.001^*^
Gender (male/female)	29/293	4/60	0.519	0.471
Disease course (month)	48[12,108]	60[12,159]	−1.537	0.124
BMI (kg/m^2^)	21.37[19.53,23.42]	20.82[19.15,22.67]	−1.298	0.194
WBC (×10^9^/L)	5.25[3.86,6.91]	5.07[3.36,7.34]	−0.785	0.433
HGB (g/L)	113[101,128]	102[89,121]	−3.449	0.001^*^
PLT (×10^9^/L)	160[114,212]	166[126,236]	−1.023	0.306
ESR (mm/h)	37[20,56]	50[26,73]	−2.652	0.008^*^
Cr (μmol/L)	52.4[44.6,63.5]	51.3[43.4,66.6]	−0.312	0.755
Urea (mmol/L)	5.32[4.22,6.75]	5.38[4.04,6.60]	−0.247	0.805
24hUpr (g/24 h)	0.24[0.08,0.91]	0.68[0.26,2.81]	−3.057	0.002^*^
LDH (U/L)	211[184,261]	274[203,341]	−4.340	<0.001^*^
CK (U/L)	41[28,64]	66[37,112]	−4.159	<0.001^*^
IgG (g/L)	15.4[11.5,19.9]	16.2[10.7,25.2]	−1.309	0.191
IgA (g/L)	2.71[1.99,3.56]	2.63[2.11,3.39]	−0.315	0.753
IgM (g/L)	0.93[0.62,1.34]	1.05[0.62,1.44]	−0.926	0.354
C3 (g/L)	0.71[0.5,0.9]	0.72[0.51,0.98]	−0.681	0.496
C4 (<0.1 g/L)	136(44%)	24(40%)	0.329	0.566
IgE (>100 IU/mL)	111(36.8%)	19(31.1%)	0.694	0.405

BMI, body mass index; WBC, white blood cell; HGB, hemoglobin; PLT, platelet; ESR, erythrocyte sedimentation rate; Cr, creatinine; 24hUpr, 24-h urinary protein quantity; LDH, lactic dehydrogenase; CK, creatine kinase; Ig, immunoglobulin; C, complement.

^*^p < 0.05 was considered statistically significant.

### The levels of serum TMs

The serum levels of CEA, CA125, CA15-3, CA19-9, SCC, CYFRA21-1, and FER were significantly elevated in the SLE-ILD group compared to the SLE group (*p* < 0.05). However, there was no significant difference in the level of NSE between the two groups (Table 2).

**Table 2:** The differences of serum TMs between the SLE-ILD group and the SLE group.

	SLE (*n* = 322)	SLE-ILD (*n* = 64)	Z	*P*
CEA (ng/mL)	1.02[0.67,1.59]	1.53[1.13,2.41]	−4.588	<0.001^*^
CA125 (U/mL)	14.7[10.5,19.93]	23.3[13.4,35.6]	−5.018	<0.001^*^
CA15-3 (U/mL)	9.2[7.1,12.3]	14.8[9.2,22.35]	−5.29	<0.001^*^
CA19-9 (U/mL)	5.14[2.66,10.17]	9.72[3.14,21.76]	−3.344	0.001^*^
SCC (ng/mL)	0.5[0.3,0.7]	0.6[0.4,0.8]	−2.164	0.03^*^
CYFRA21-1 (ng/mL)	1.14[0.86,1.79]	1.81[1.27,2.35]	−4.315	<0.001^*^
NSE (ng/mL)	14.54[11.39,19.28]	15.72[12.69,20.82]	−1.5	0.134
FER (ng/mL)	142.26[58.81,257.41]	194.24[88.12,386.08]	−2.2	0.028^*^

CEA, carcinoembryonic antigen; CA, carbohydrate antigen; SCC, squamous cell carcinoma antigen; CYFRA21-1, cytokeratin 19 fragment; NSE, neuron-specific enolase; FER, ferritin.

^*^p < 0.05 was considered statistically significant.

### Risk factors of SLE-ILD

**Figure 2:**
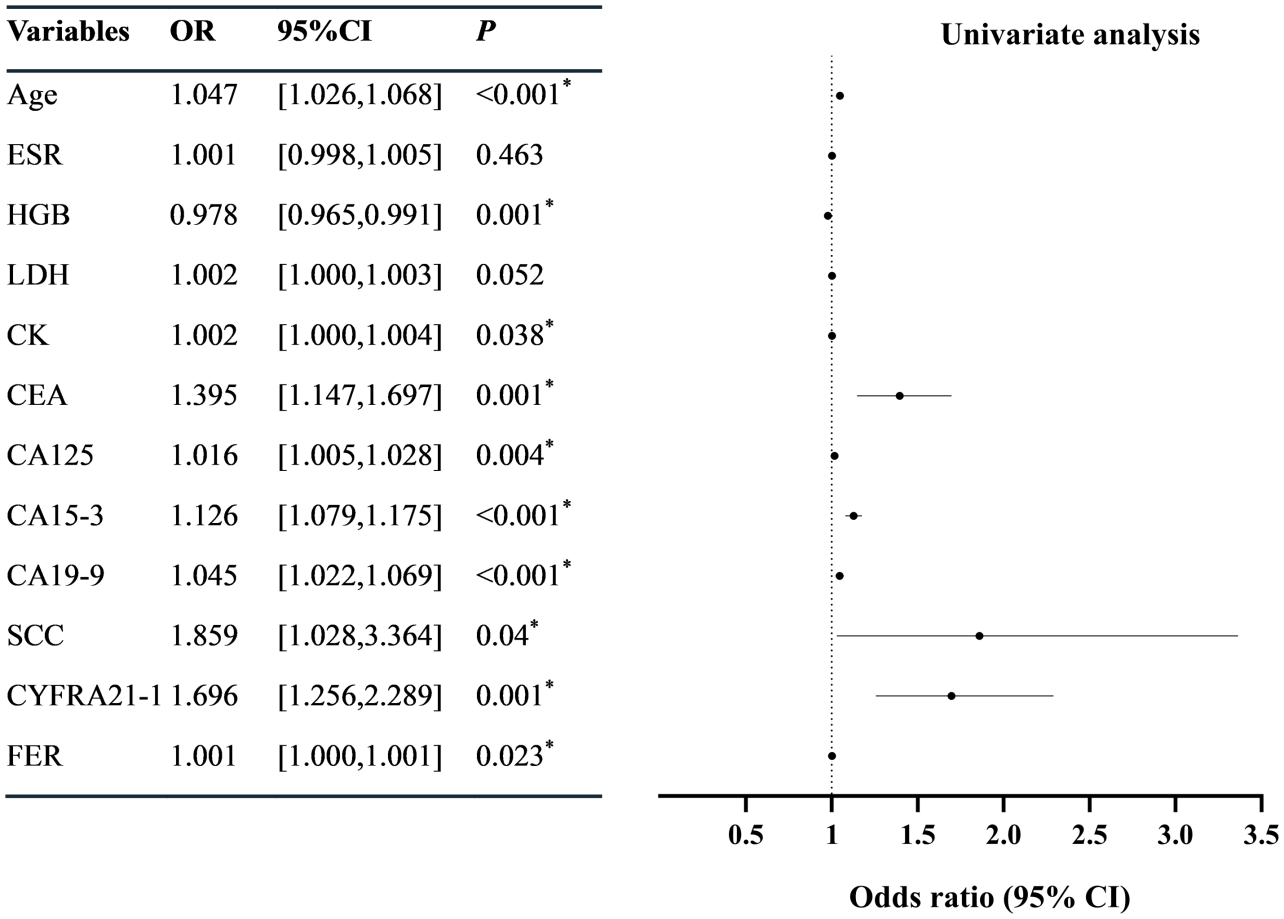
Univariate logistic regression analysis of risk factors in patients with SLE-ILD. ESR, erythrocyte sedimentation rate; HGB, hemoglobin; LDH, lactic dehydrogenase; CK, creatine kinase; CEA, carcinoembryonic antigen; CA, carbohydrate antigen; SCC, squamous cell carcinoma antigen; CYFRA21-1, Cytokeratin 19 fragment; NSE, neuron-specific enolase; FER, ferritin. ^*^p < 0.05 was considered statistically significant.

**Figure 3:**
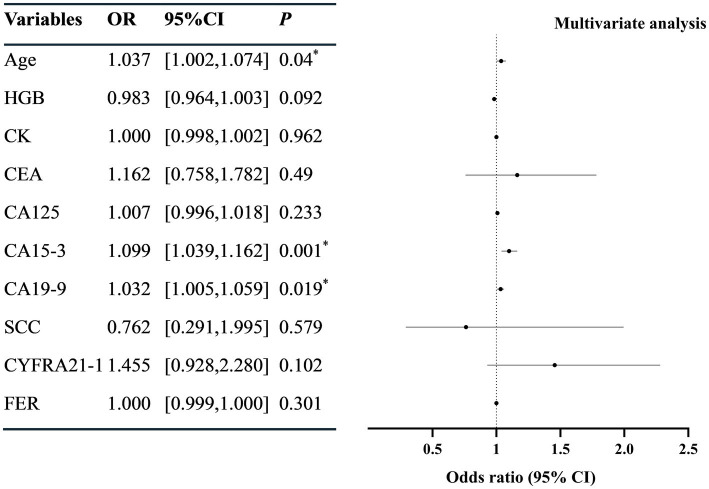
Multivariate logistic regression analysis of risk factors in patients with SLE-ILD. HGB, hemoglobin; CK, creatine kinase; CEA, carcinoembryonic antigen; CA, carbohydrate antigen; SCC, squamous cell carcinoma antigen; CYFRA21-1, cytokeratin 19 fragment; NSE, neuron-specific enolase; FER, ferritin. ^*^p < 0.05 was considered statistically significant.

Univariate Logistic regression revealed that the age, serum CK, CEA, CA125, CA15-3, CA19-9, SCC, CYFRA21-1, and FER were correlated with SLE-ILD (*p* < 0.05) (Figure 2). The collinearity diagnostic results indicated no collinearity among the variables (Supplementary Table 2). Multivariate Logistic regression analysis identified age (OR = 1.038, 95%CI = [1.004, 1.074]), CA15-3 (OR = 1.099, 95%CI = [1.039, 1.162]), and CA19-9 (OR = 1.032, 95% CI = [1.005, 1.059]) as independent risk factors for SLE-ILD (*p* < 0.05) (Figure 3).

### Diagnostic values of TMs

**Figure 4:**
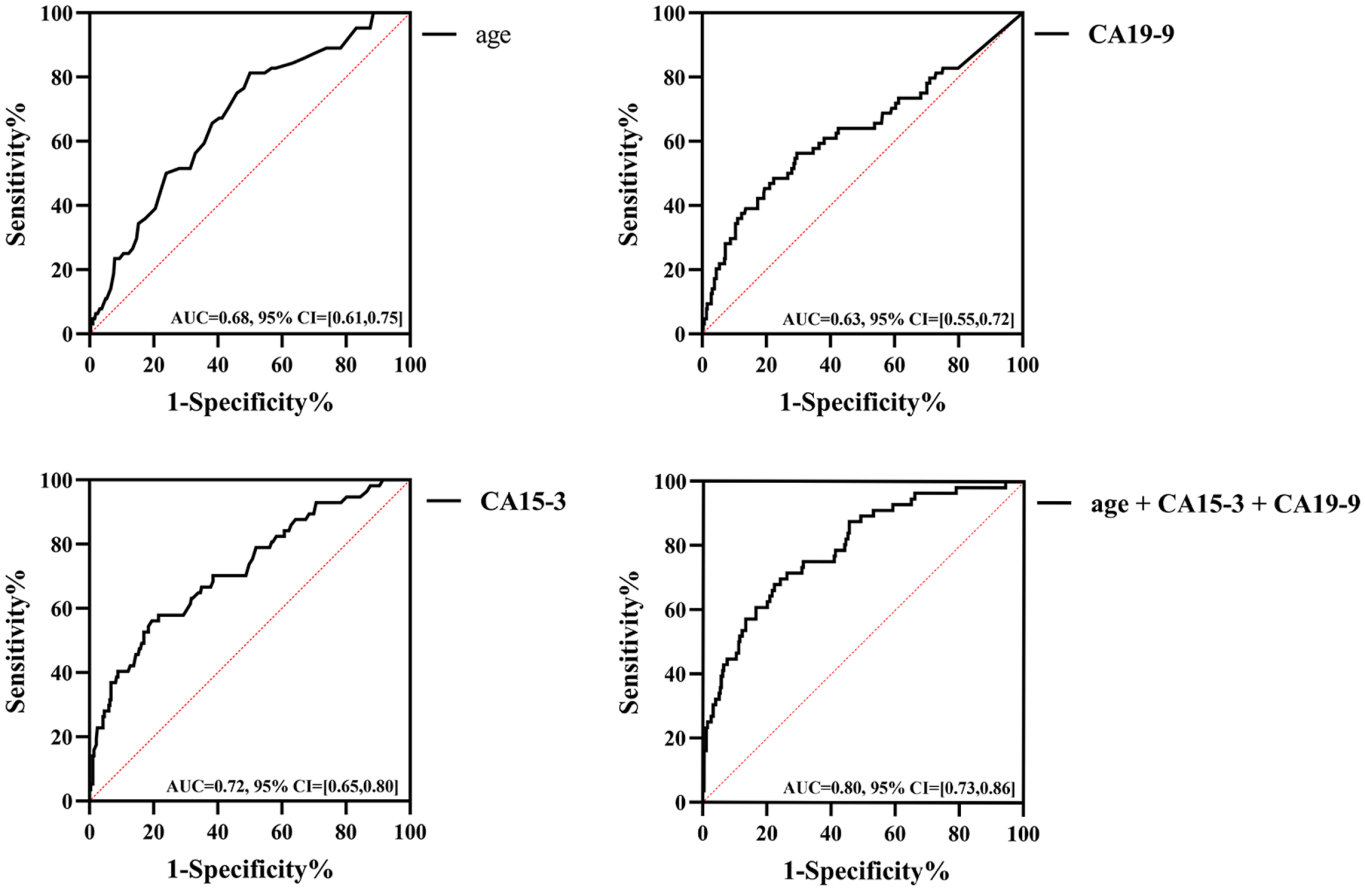
Diagnostic efficacy of age, CA15-3, CA19-9 and three combinations in patients with SLE-ILD.

The ROC curves showed that the AUC for age was 0.68 (95% CI = [0.61, 0.75], *p* < 0.05), with a cut-off value of 37.5, sensitivity of 81.3%, and specificity of 50%; the AUC for CA15-3 was 0.72 (95% CI = [0.65, 0.80], *p* < 0.05), with a cut-off value of 13.8 U/mL, sensitivity of 56.1%, and specificity of 80.1% (Figure 4). For CA19-9, the AUC was determined to be 0.63 (95% CI = [0.55, 0.72], *p* < 0.05), with a cut-off value set at 8.75 U/mL; its sensitivity was 56.3%, and specificity was 70.4%. The combined AUC for the three indicators was 0.8 (95% CI = [0.73–0.86], *p* < 0.001); the sensitivity and specificity were 68.4 and 76%, respectively. Diagnostic values of other TMs (CEA, CA125, SCC, CYFRA21-1 and FER) were shown in Supplementary Table 3.

### Correlation analysis of TMs and the involvement of SLE-ILD in other organs

For the TMs, the CEA level demonstrated a low positive correlation with both HGB and Urea levels (*r* = 0.262 and 0.3 respectively, *p* < 0.05). Additionally, SCC and CYFRA21-1 levels showed a moderate positive correlation with Cr levels (*r* = 0.562 and 0.713 respectively, *p* < 0.001). Furthermore, the FER level exhibited a low positive correlation with blood WBC and Cr levels (*r* = 0.248 and 0.489 respectively, *p* < 0.05) (Table 3).

**Table 3:** Pearson correlation of TMs and blood cell analysis and kidney function in patients with SLE-ILD.

	WBC	HGB	PLT	Urea	Cr	24hUpr
CEA	0.191	0.262^*^	−0.22	0.3^*^	0.159	−0.122
CA125	0.216	−0.117	0.059	0.041	0.042	0.024
CA15-3	0.199	−0.096	−0.058	0.014	0.002	0.012
CA19-9	0.245	0.101	−0.081	0.202	0.053	0.319
SCC	−0.039	−0.039	−0.166	0.141	0.562^**^	0.244
CYFRA21-1	0.028	−0.13	−0.102	0.24	0.713^**^	0.129
FER	0.248^*^	0.008	0.067	0.062	0.489^**^	−0.046

WBC, white blood cell; HGB, hemoglobin; PLT, platelet; Cr, creatinine; 24hUpr, 24-h urinary protein quantity; CA, carbohydrate antigen; SCC, squamous cell carcinoma antigen; CYFRA21-1, cytokeratin 19 fragment; FER, ferritin.

^*^p < 0.05, ^**^p < 0.01.

## Discussion

Exploring biomarkers is crucial for the early identification of ILD, but the diagnostic efficacy may vary across different types of ILD. Given that ILD encompasses a collection of heterogeneous diseases, it is necessary to identify potential biomarkers for different ILD subtypes. In recent years, serum TMs have emerged as candidate markers for detecting CTD-ILD (12). Studies on the relationship between RA-ILD, primary Sjögren’s syndrome-associated interstitial lung disease (pSS-ILD), and dermatomyositis-associated interstitial lung disease (DM-ILD) with TMs indicated that TMs provided good diagnostic value in evaluating the disease status of CTD-ILD (7, 8, 13). Our study showed the clinical and laboratory parameters from patients with SLE-ILD, thereby providing clinical evidence for the role of TMs in assessing SLE-ILD.

According to the findings of our study, patients with SLE-ILD were older than those without ILD. Furthermore, the risk of ILD increased with age, suggesting that age played an important role in causing pulmonary fibrosis. This observation is consistent with a study by Liu et al. (14). Additionally, the levels of ESR and LDH in patients with SLE-ILD were significantly higher than those in patients without ILD. Elevated ESR and LDH levels are also noted in patients with SLE-associated pleurisy (15), and both are considered to be associated with the activity of inflammatory states. These findings suggest ESR and LDH may be potential indicators of lung involvement in SLE.

Serum TMs are important indicators in clinical laboratories, playing a significant role in tumor proliferation, invasion, and metastasis (16). In addition to the association with tumors, TMs also function as inflammatory factors that are of great importance in assessing the degree of inflammation, disease activity, and organ involvement in CTD (9, 12). It is noteworthy that serum TMs levels may be influenced by various confounding factors. Wang et al. confirmed elevated SCC levels in the peripheral blood of patients with community-acquired pneumonia (17), while serum CA125 showed diagnostic significance for tuberculosis infection (18). In addition to infectious diseases, some gastrointestinal diseases such as acute cholecystitis and acute appendicitis could be accompanied by increased serum TMs (19, 20). Furthermore, since SLE predominantly occurs in women of childbearing age, pregnancy also made an impact on serum TMs levels (21). To sum up, we ruled out some confounding factors to ensure the reliability of the results.

There exists a relationship between tumor marker levels and disease state in ILD. For example, among patients with CTD-ILD, elevated CEA and CA19-9 levels indicated severe disease (22); higher CA19-9 levels correlated with poorer lung function within ILD patients (23). In cases involving DM-ILD, when CEA and FER levels rose, there was a risk of rapid ILD progression (24). In patients with ILD, higher CA19-9 levels signified poorer lung function, and higher CA15-3 levels correlated with more severe interstitial lung lesions (25, 26). These results underscored the significant heterogeneity in serum TMs levels for evaluating different types of CTD-ILD, highlighting the necessity for exploring the diagnostic capability of TMs across various CTD-ILD subtypes.

Our study showed that CA15-3 and CA19-9 levels provided good diagnostic values for SLE-ILD. Due to heterogeneity, serum CA15-3 and CEA levels in other types of CTD-ILD were believed to be involved in the occurrence of Sjögren’s syndrome-associated ILD (7), Sargin G et al. (27) found that levels of serum CA125, CA153 and CA199 in patients with RA-ILD were significantly higher than those in RA patients without ILD; they proposed TMs as predictive biomarkers for RA-ILD. A subsequent study by Zheng et al. (28) further confirmed that CA19-9 was an important risk factor for RA-ILD. Bao et al. (12) reported that increased levels of CA15-3 and CYFRA21-1 signified a high risk for CTD-ILD. It may be owing to various factors such as pathophysiological mechanisms and inflammatory response pathways when different types of CTD invaded lung tissues. However, Zhong et al. (29) observed elevated serum TMs levels in patients with SLE-related serous effusion and proposed that this elevation may be attributed to inflammation associated with SLE. It is important to note that serum TMs could be influenced by various factors. Therefore, a biomarker with high diagnostic value for SLE-ILD is still unavailable. Although Krebs Von den Lungen-6, surfactant protein-D and interleukin 6 exhibit certain diagnostic values in ILD patients (30–32), no single TM can fulfill the necessary requirements of sensitivity and specificity in diagnosing SLE-ILD. It is recommended that future research should explore the combination of serum TMs with other biomarkers to enhance the diagnostic efficacy of serum TMs. A study by Wan et al. (33) clarified that combining TMs, inflammatory factors and disease activity indicators could markedly improve the diagnostic capability for RA-ILD. Our results showed that the combination of age, CA15-3 and CA19-9 could significantly increase the diagnostic value of SLE-ILD. These indicated that combining multiple biomarkers or clinical indicators can better improve the diagnostic accuracy of disease status and prognosis for SLE-ILD.

Serum TMs may also serve as signaling molecules for disease activity and organ involvement in SLE. Szekanecz et al. (9) verified that the increased serum levels of CEA, CA15-3, and CA19-9 in patients with SLE correlated with SLE-associated kidney impairment. However, in our study, elevated CEA levels were positively associated with Urea levels in patients with SLE-ILD, while no correlation was observed between serum CA15-3 and CA19-9 levels and kidney impairment. It may be due to our focus on patients with SLE-ILD, without separately investigating those experiencing SLE-related kidney impairment. Moreover, our study proved for the first time that serum SCC, CYFRA21-1 and FER levels in patients with SLE-ILD were moderately positively correlated with serum Cr levels. Although previous studies indicated no relationship between SCC and SLE activity (34), they did not elucidated whether SCC or other markers were involved in kidney impairment in SLE, nor did they address the correlation between serum TMs and organ damage in SLE apart from the kidneys. It is worth mentioning that Miret et al. (34) also indicated a correlation between serum TMs and renal function impairment in SLE; however, they were unable to elucidate the potential mechanisms underlying this association. We propose that this may be linked to the diminished metabolic capacity of TMs following kidney injury. Previous studies have shown that levels of serum SCC and CYFRA21-1 in patients with chronic kidney disease are positively correlated with Cr (35). Conversely, further research is needed to determine whether elevated TMs contribute to renal involvement in SLE-ILD patients, as well as to explore the underlying pathophysiological mechanisms involved.

There are still some limitations. Firstly, the correlations between serum TMs and the severity and prognosis of SLE-ILD have not been elucidated. In addition, due to the variations in serum TMs levels associated with malignant or benign diseases, our study did not investigate differences in serum TMs levels and clinical significance across other lung cancers, other CTD or ILD populations. Furthermore, given the limitation of retrospective designs, future prospective studies with larger sample sizes are necessary to account for the influence of various factors on the outcomes of SLE-ILD and TMs. These factors include such as inclusion criteria, follow-up duration, ILD diagnosis, TMs detection methods, and drug interventions. Such efforts will be essential for further exploring the role of serum TMs in assessing SLE-ILD disease status. Moreover, future research should also consider comparing serum TMs with other markers such as Krebs Von den Lungen-6, surfactant protein-D and interleukin 6 to better understand the diagnostic value of serum TMs in SLE-ILD. Additionally, joint analyses involving both TMs and these biomarkers may be beneficial for improving the diagnostic effectiveness of SLE-ILD. Secondly, the relationship among serum TM levels, SLE disease activity and HRCT imaging features in patients with SLE-ILD has not been clarified. It is necessary to investigate the underlying reasons for the association between TMs and organ impairment, particularly in the kidney, as it is associated with SLE-ILD in future studies. Finally, large-scale multicenter prospective cohort studies should be conducted to explore the relationship between serum TMs and other organ impairments affected by SLE, including the nervous system, gastrointestinal tract, cardiovascular and joints.

In conclusion, our study demonstrated that monitoring serum TMs was clinically significant in SLE-ILD. Additionally, age as well as serum CA153 and CA19-9 levels may serve as factors associated with SLE-ILD. Besides, serum SCC, CYFRA21-1, and FER levels may be linked to kidney involvement in patients with SLE-ILD.

## Data Availability

The raw data supporting the conclusions of this article will be made available by the authors, without undue reservation.
