# Supplementary material for: A correlation study of serum tumor markers with systemic lupus erythematosus-associated interstitial lung disease
Source: Front Med (Lausanne). 2025 Mar 18;12:1558702. doi: 10.3389/fmed.2025.1558702 (PMC11959046; doi:10.3389/fmed.2025.1558702)
Supplement: Supplementary file 1 [file Data_Sheet_1.docx]

**Supplementary Table 1 Clinical features of 64 patients with SLE-ILD included**

|  | SLE-ILD |
| --- | --- |
| **SLE-related antibodies** |  |
| Anti-U1-RNP |  |
| (-) | 24(37.5) |
| (+) | 5(7.8) |
| (++) | 1(1.6) |
| (+++) | 34(53.1) |
| Anti-Sm |  |
| (-) | 45(70.3) |
| (+) | 7(10.9) |
| (++) | 4(6.3) |
| (+++) | 8(12.5) |
| Anti-Ro (SSA) |  |
| (-) | 23(35.9) |
| (+) | 11(17.2) |
| (++) | 5(7.8) |
| (+++) | 25(39.1) |
| Anti-Ro52 |  |
| (-) | 29(45.3) |
| (+) | 4(6.3) |
| (++) | 5(7.8) |
| (+++) | 26(40.6) |
| Anti-La (SSB) |  |
| (-) | 59(92.2) |
| (+) | NA |
| (++) | 4(6.3) |
| (+++) | 1(1.6) |
| Anti-CENP-B |  |
| (-) | 59(92.2) |
| (+) | 1(1.6) |
| (++) | NA |
| (+++) | 4(6.3) |
| Anti-PCNA |  |
| (-) | 61(95.3) |
| (+) | 3(4.7) |
| (++) | NA |
| (+++) | NA |
| Anti-dsDNA |  |
| (-) | 43(67.2) |
| (+) | 9(14.1) |
| (++) | 7(10.9) |
| (+++) | 5(7.8) |
| ANuA |  |
| (-) | 33(51.6) |
| (+) | 12(18.8) |
| (++) | 12(18.8) |
| (+++) | 7(10.9) |
| AHA |  |
| (-) | 34(53.1) |
| (+) | 12(18.8) |
| (++) | 12(18.8) |
| (+++) | 6(9.4) |
| Anti-Rib-P |  |
| (-) | 42(65.6) |
| (+) | 5(7.8) |
| (++) | 2(3.1) |
| (+++) | 15(23.4) |
| ANA titer |  |
| 1:100 | 8(12.5) |
| 1:320  1:1000 | 6(9.4) |
|  | 10(15.6) |
| 1:3200 | 37(57.8) |
| 1:10000 | 3(4.7) |
| **HRCT imaging findings** |  |
| NSIP | 39(60.9) |
| UIP | 13(20.3) |
| OP | 1(1.6) |
| LIP | 3(4.7) |
| NSIP overlap OP | 8(12.5) |

NA: not available; ANuA: anti-nucleosome antibody; AHA: anti-histone antibody; ANA: antinuclear antibody; HRCT: high-resolution computed tomography; NSIP: nonspecific interstitial pneumonia; UIP: usual interstitial pneumonia; OP: organizing pneumonia; LIP: lymphocytic interstitial pneumonia. NA: not available

**Supplementary Table 2 Collinearity diagnosis of independent variables in multiple logistic regression analysis**

| model | Collinearity Statistics | |
| --- | --- | --- |
|  | Tolerance | VIF |
| (constant) |  |  |
| age | 0.730 | 1.369 |
| HGB | 0.929 | 1.077 |
| CK | 0.880 | 1.136 |
| CEA | 0.619 | 1.616 |
| Ca125 | 0.957 | 1.045 |
| Ca153 | 0.891 | 1.122 |
| Ca199 | 0.876 | 1.142 |
| SCC | 0.879 | 1.137 |
| CYRFA211 | 0.755 | 1.325 |
| FER | 0.905 | 1.105 |

HGB: hemoglobin; LDH: lactic dehydrogenase; CK: creatine kinase; CEA: carcinoembryonic antigen; CA: carbohydrate antigen; SCC: squamous cell carcinoma antigen; CYFRA21-1: Cytokeratin 19 fragment; NSE: neuronspecific enolase; FER: ferritin; VIF: variance inflation factor.

**Supplementary Table 3 Diagnostic values of CEA, CA12-5, SCC, CYFRA21-1 and FER in SLE-ILD**

|  | **CEA** | **CA125** | **SCC** | **CYFRA21-1** | **FER** |
| --- | --- | --- | --- | --- | --- |
| **AUC** | 0.68 | 0.7 | 0.6 | 0.69 | 0.59 |
| **95%CI** | [0.61,0.77] | [0.63,0.77] | [0.51,0.68] | [0.62,0.77] | [0.51,0.67] |
| ***P*** | <0.001 | <0.001 | 0.031 | <0.001 | 0.028 |
| **Cut-off value** | 1.13(ng/mL) | 22.05(U/mL) | 0.75(ng/mL) | 1.23(ng/mL) | 192.73(ng/mL) |
| **Sensitivity(%)** | 0.762 | 0.561 | 0.4 | 0.78 | 0.54 |
| **Specificity(%)** | 0.563 | 0.811 | 0.77 | 0.569 | 0.625 |

AUC: area under the ROC curve; CI: confidence interval; CEA: carcinoembryonic antigen; CA: carbohydrate antigen; SCC: squamous cell carcinoma antigen; CYFRA21-1: Cytokeratin 19 fragment; FER: ferritin.

*P*<0.05 denotes statistically significant.
